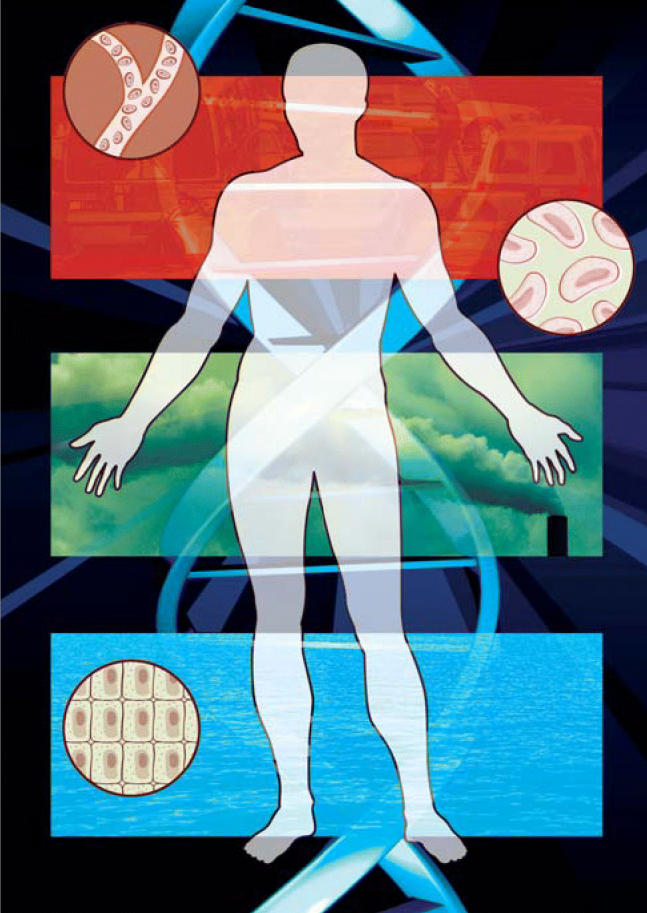# Gene–Environment Studies: Who, How, When, and Where?

**DOI:** 10.1289/ehp.114-a466

**Published:** 2006-08

**Authors:** Angela Spivey

With the sequencing of the human genome completed, the question becomes: what now? Many common diseases are known to be associated with genetic variants, or changes in single nucleotides of the DNA making up the human genome. However, scientists still have many questions about how individual gene variants, and interactions between variants and environmental factors, contribute to an individual’s risk of developing common diseases such as cancer, obesity, and heart disease.

Some scientists believe the only way to answer those questions is through a large prospective cohort study, collecting DNA samples and information about exposure to a variety of environmental factors from 500,000 to 1 million participants and following this random sampling of the population over a number of years. But such a study would require a huge investment of time, effort, and money; the DHHS Secretary’s Advisory Committee on Genetics, Health, and Society (SACGHS) estimates the cost at roughly $3 billion, possibly more. In addition, such an endeavor would likely raise significant social, legal, and ethical issues concerning privacy, consent, public involvement, and communication.

Now a new draft report by the SACGHS examines the policy issues related to such a study. The report concludes that, although conducting a large prospective study presents major challenges, it also has the potential to result in significant health benefits.

## Examining the Angles

In 2004 the SACGHS decided to address the question of whether the United States should undertake a large cohort study in this country. The committee formed the Large Population Studies Task Force to dig into the issues that would be involved in such a study. Since a large population project could potentially have significant ethical, regulatory, scientific, and public health implications, NIH director Elias A. Zerhouni asked the committee to focus its inquiry on the associated policy issues.

Through consultation with experts in the field, fact-finding research, and deliberation, the committee identified several specific policy issues. In May 2006, the committee issued a draft report (available at http://www4.od.nih.gov/oba/SACGHS/public_comments.htm) that discussed these key policy issues and made recommendations for how they might be addressed. The report was then opened to the public for comment through the end of July 2006.

The report devotes an entire chapter to the need for public involvement in all stages of the decision making, planning, and execution of such a study. Suggested populations to consult include the scientific and international communities, representatives of populations that might be involved in the research, health care providers and their institutions, and those who volunteer to participate in the project as research subjects. The report also stresses the need to include in the study populations who are underinsured or who are underserved by the health care system. Since such a study would require a large investment of public money, states the report, it is only reasonable and fair that the benefits should be equitably distributed among the population.

## Honing the Tools

The report notes that some scientists raise the question of whether scientific methods to determine gene–environment interactions are mature enough to obtain maximum value from a large prospective study. Current methods of measuring exposures allow scientists to determine that an environmental exposure is correlated with disease, but it is still difficult to understand the mechanisms underlying such associations, said NIEHS director David Schwartz during a June 2006 presentation to the SACGHS.

Schwartz is co-chairman, with National Human Genome Research Institute director Francis Collins, of the NIH Coordinating Committee for the Genes and Environment Initiative, a just-launched research effort that aims to develop more precise tools that could be useful in a large cohort study. Tools such as biological sensors and biomarkers would allow scientists to determine not just what a person has been exposed to, but whether the person’s body is responding to an exposure, Schwartz said during his presentation.

Other concerns focus on issues of study design. John Hewitt, director of the Institute for Behavioral Genetics and a professor of psychology at the University of Colorado, also made a presentation before the SACGHS in June 2006. Hewitt suggested that the committee consider highlighting the need for a smaller substudy of identical twins, which could serve to confirm apparent associations between disease and either environmental factors or gene–environment interactions.

“The big concern is that a large-scale national study has a very wide geographic and demographic range, so it’s very difficult to sort out what are truly environmental differences and what are truly genetic differences,” Hewitt says. “When you study genetically identical pairs, you know that the environmental differences within that pair aren’t correlated with genetic differences, because there are [no genetic differences].”

A substudy of twins may also help keep the larger study honest. “You could certainly take things that appear to be interesting in the large study and get an immediate check [in a twin study] on whether those environmental associations held up when you controlled for the genotype,” Hewitt says.

## How and When to Return Results

Richard Sharp, an assistant professor of medicine with the Center for Medical Ethics and Health Policy at Baylor College of Medicine, praises the report’s commitment to reaching out to the public and to underrepresented communities. But he expresses surprise that the report didn’t pay more attention to what he calls “relatively obvious” ethical issues surrounding informed consent and communicating research results to participants.

“If you’re a patient in a clinic in a hospital, and someone comes up to you and says ‘we want to enroll you in this twenty-year study,’ or however long it ends up being, what would you need to know before you felt like you could say yes or no?” Sharp asks. He and NIEHS health administrator Pat Chulada conducted a study with participants in the NIEHS’s Environmental Polymorphisms Registry to answer these questions. The data from that study are now being analyzed.

Sharp also stresses the importance of establishing a process for communicating research results to participants. For instance, if certain genes are found to greatly increase risk for certain diseases, should study participants be informed about these results and their genetic status? If so, when and how? The report suggests that any large prospective study should include a standing committee to address such ethical issues but doesn’t outline a specific process for returning results. “We don’t really know what to do in terms of returning results of unclear value,” Sharp says.

Task force chairman Huntington F. Willard, director of the Institute for Genome Sciences & Policy at Duke University, emphasizes that the draft report has not yet been approved by the full committee. With the comment period now completed, the task force will consider the comments, modify the draft, and present it to the SACGHS for its consideration and action, most likely at the committee’s November 2006 meeting. However, even as many scientists express enthusiasm for the benefits of such a study, the SACGHS and others will still need to explore the many other challenges to be addressed, not the least of which is the uncertain availability of funding.

## Figures and Tables

**Figure:**